# On the Mutual Relations between Anatomy, Pathology, Physiology, and Therapeutics and the Practice of Medicine

**Published:** 1842-11-26

**Authors:** 


*On the Mutual Relations between Anatomy, Pathology, Physiology, and Therapeutics and the Practice of Medicine. Being the Gulstonian Lectures for* 1842. By Marshall Hall, M. D., F. R. S. L. and E., Fellow of the Royal College of Physicians, &c. &c. Illustrated by three engraved Plates. London: 1842. 8vo. p. 86.

Dr. Hall thinks that a strong prejudice exists, both in the public mind and in that of the profession, against the study of anatomy and physiology. It is mentioned by the biographer of the celebrated Dr. Baillie that his friends feared that his prëeminence in anatomical knowledge, instead of establishing his fame as a practitioner, would absolutely not only impede but frustrate his prospects. To remove a prejudice so unjust, and so injurious to medical science, is the object of the present lectures; and this is to be done "by demonstrating that the physiologist alone can become the truly able practitioner—that *science* alone, properly pursued and applied, and not what has been fallaciously and, I fear, too often insidiously denominated *experience*, can lead to a just diagnosis, unfold the nature of disease, suggest new remedies, and guide us in the just employment of the old," (p. 2.) Numerous examples might be adduced of the most eminent physicians formed in anatomical and physiological schools: Hunter, Heberden, Baillie, Sir Benjamin Brodie, our author himself, and a host of others equally distinguished. "Every day physiology becomes a more certain science, and more a science of phenomena and principles. Theory is taking the place of hypothesis; experiment and observation, of conjecture. Every day, too, the bond which unites the science of physiology and the practice of medicine is drawn tighter," (p. 8.) A treatise on the subject of these lectures, in which each principle is illustrated by a fact,—an observation or an experiment,—would be of incalculable benefit. Such a treatise, Dr. Hall informs us, he has projected and still expects to execute, in which hope we fervently join.

The principal subjects treated of in these lectures, are:

1. The comparative importance of ingestion and egestion in a physiological and pathological point of view. 2. The application of the principle of the diffusion of gases to the physiology of respiration, first developed by Professor Graham; and to the pathology of this function, or asphyxia. 3. The single, but diplo-cardiac circulation in man, with its application to the pathology of the "arrière" (?) circulation in certain diseases. 4. The coronary circulation, as implying the necessity of the conjunction of the systemic and pulmonic hearts, and as explaining the occurrence of certain cases of sudden death. 5. The condition of the blood in asphyxia, anemia, chlorosis, euthismus, &c., and in relation to sudden death. 6. The two-fold method of operation of remedies and poisons, and of both internal and external agents generally, in different instances, through the *nervous* and *vascular* systems.

These lectures strike us as rather elementary for the character of the audience before whom they were delivered, and, though containing certainly a share of the acute thought and enlightened experience of the eminent author, have much also that is trivial and unsound—much hasty and unwarranted generalization. The introduction of whole pages of French must frequently have proved rather puzzling and wearisome to his auditory, and is of very questionable taste.

